# Analysis of Signals Leading to Differentiation of Immature HL-60 after Administration of IL-4

**DOI:** 10.1155/S096293519400058X

**Published:** 1994

**Authors:** Simon Vassiliadis

**Affiliations:** University of Crete Department of Biology P.O. Box 1470 Crete Heraklion 711-10 Greece

## Abstract

Treatment of acute myeloid leukaemia (AML) cells with differentiation agents leads not only to the acquisition of normal phenotypes but also contributes to the understanding of special immuno-haematology issues. For instance, induction of HLA-DR antigens on human promyelocytic leukaemia HL-60 cells by interleukin-4 (IL-4) is of pivotal importance in immunology not only because class II expression is prerequisite to antigen recognition and response but also because IL-4 participates in a plethora of inflammatory or non-inflammatory reactions. At the same time, the same observation coupled with an increase in Mac-1, mature monocyte marker, is revealing ways to haematologists for converting malignant cases to normal situations. Based on previous reports that HLA-DR induction by IL-4 in the HL-60 system is mediated via the G-protein system (p21^ras^), this study was undertaken in order to define the intermediate signalling steps followed by this agent from the moment it is added to cultures to the differentiated cellular form obtained. It is proposed that IL-4 increases p21^ras^ which in turn suppresses the HL-60 cells' p34^cdc2^ constitutive expression. This inhibition appears to be responsible for the subsequently.observed cessation ofgrowth. Concomitant to decreased cellular proliferation, HI-DR antigen expression increases, a finding that matches the initially mentioned induction of p21^ras^ since its inhibition abolishes HI-DR upregulation.


Mediators of Inflammation 3, 415–418 (1994)

### Analysis of signals leading to differentiation of immature HL-60 after administration of IL-4

Simon Vassiliadis

University of Crete, Department of Biology, P.O. Box 1470, Heraklion 711-10, Crete, Greece

**Treatment of acute myeloid leukaemia (AML) cells with differentiation agents leads not only to the acquisition of normal phenotypes but also contributes to the understanding of special immuno-haematology issues. For instance, induction of HLA-DR antigens on human promyelocytic leukaemia HL-60 cells by interleukin-4 (IL-4) is of pivotal importance in immunology not only because class II expression is prerequisite to antigen recognition and response but also because IL-4 participates in a plethora of inflammatory or non-inflammatory reactions. At the same time, the same observation coupled with an increase in Mac-1, mature monocyte marker, is revealing ways to haematologists for converting malignant cases to normal situations. Based on previous reports that HLA-DR induction by IL-4 in the HL-60 system is mediated via the G-protein system ($p21^{ras}$), this study was undertaken in order to define the intermediate signalling steps followed by this agent from the moment it is added to cultures to the differentiated cellular form obtained. It is proposed that IL-4 increases $p21^{ras}$ which in turn suppresses the HL-60 cells' $p34^{cdc2}$ constitutive expression. This inhibition appears to be responsible for the subsequently observed cessation of growth. Concomitant to decreased cellular proliferation, HLA-DR antigen expression increases, a finding that matches the initially mentioned induction of $p21^{ras}$ since its inhibition abolishes HLA-DR upregulation.**

**Key words:** Differentiation, HL-60, HLA-DR, IL-4, $p21^{ras}$, $p34^{cdc2}$, Proliferation

### Introduction

IL-4 is a T-cell derived factor[1,2] exerting its biological actions mainly on B-cells.[3] However, the presence of the IL-4 receptor is not restricted to the B-cell lineage but also found on a variety of other cells and cell lines, one of which is the human promyelocytic leukaemia HL-60 cell line.[4] This line was derived from an acute myeloid leukaemia (AML) patient with cells arrested at the blast stage.[5] Terminal differentiation of HL-60 cells can be achieved by external stimuli like chemical components or biological agents (for review see Collins[6]). The obvious advantage of physiological agents vis-à-vis chemicals led this investigation to the use of IL-4 since this line expresses IL-4 receptors ectopically.[4,7] It has been previously shown that IL-4 is able to initiate a maturation programme and lead the immature, class II negative HL-60 cells to a differentiated state expressing HLA–DR surface antigens.[8] These antigenic determinants were shown to be mediated via the G-protein system since $p21^{ras}$ induction was shown to be concomitant to class II after IL-4 administration.[8] More recent reports in the *Xenopus* system claimed that oncogenic *ras* may block cell cycle progression and inhibit $p34^{cdc2}$ kinase activity[9] by a mechanism other than blocking cyclin B synthesis or formation of the cyclin B–$p34^{cdc2}$ complex,[9] both absolutely necessary for a cell to progress through mitosis.[10–12]

Since HL-60 has a high and abnormal proliferative rate,[5] one may hypothesize that this unlimited growth behaviour is due to the presence of the cdc2 gene product, p34, as it happens to other similar cells and cell lines.[13] Once this question was answered positively (this work) and in conjunction with the ability IL-4 has to induce $p21^{ras}$ in the same system,[8] an effort was made to put the pieces of the puzzle together and follow all the possible intracellular signals from the moment IL-4 comes in contact with the cells up to the differentiated state.

It is proposed that the possible cascade of events taking place after IL-4 administration are an increase of $p21^{ras}$ which suppresses the constitutive expression of $p34^{cdc2}$ thus leading to a controlled cellular growth pattern and an increase in class II expression. Since inhibition of induced $p21^{ras}$ brings p34 and HLA-DR expressions to control levels, it is likely that the proposed sequence represents the actual situation.

© 1994 Rapid Communications of Oxford Ltd

### Materials and Methods

*Cells*: HL-60 cells[5,6] were purchased from the American Type Culture Collection (ATCC, Rockville, MD) and were grown in RPMI 1640 culture medium (Gibco, Grand Island, NY) supplemented with 10% foetal calf serum (FCS, Seralab, Sussex, UK) at 37°C, 5% $CO_2$.

*Growth factors and antibodies*: Human recombinant IL-4 was purchased from Genzyme (Boston, MA) and was used at a concentration of 200 units/ml (200 U of IL-4 contain 0.01 µg of protein). The anti-$p21^{ras}$ monoclonal antibody, a rat IgG, was purchased from Oncogene Science (Manhasset, NY) and used at a dilution of 1:100 (1.0 µg). The IgG1 anti-cdc2 monoclonal antibody was purchased from UBI (Lake Placid, NY) and used at a concentration of 10 µg/ml (1 µg test). Viability tests by Trypan blue dye exclusion were used to demonstrate that all working solutions were not toxic.

*Induction protocol*: One hundred thousand HL-60 cells were cultured with 200 U/ml IL-4 on Day 0. At 24 and 48 h, another boost of the same dose of IL-4 was administered to cells[7] and class II as well as $p34^{cdc2}$ surface expression was monitored at 72 h.

*HLA-DR analysis*: HLA-DR was monitored with the use of Dynabeads (Dynal A.S., Norway) coated with a human anti class II (DR) monoclonal antibody in one-step experiments as previously described.[8] The number of cells and beads employed was in accordance with the manufacturer's instructions.

*Indirect immunofluorescence for determining* ras *and $p34^{cdc2}$ expression*: Control and treated HL-60 cells were washed in PBS (Gibco) and placed in 96-well plates (Linbro, Flow Labs, McLean, VA) at a concentration of $5 \times 10^6$ cells/ml. The cells were incubated with PBS supplemented with 0.2% BSA (Sigma, St Louis, MO) and 0.01% sodium azide (Sigma, PBS–BSA–Azide). Before testing, the cells were incubated with 20% ice-cold methanol for 15 min to allow membrane permeabilization since the *ras* antigen is found in the inner surface membrane of the cells.[14] For $p34^{cdc2}$ detection, the ice-cold methanol treatment was omitted. Test antibodies, diluted as described above, were added to the wells (100 µl/well) and incubated for 45 min at 4°C. After washing the cells three times with PBS–BSA–Azide, FITC-conjugated goat-anti-rat or anti-mouse respectively IgG antibody (Tago Inc., Burlingame, CA) was added for 45 min at 4°C. The cells were extensively washed, fixed with 25% glycerol, and mounted on slides. Fluorescence was evaluated visually using a Zeiss (Oberkochen, Germany) fluorescent microscope. Cells with weak or no staining were scored as negative. Positive cells were considered to be those showing bright to very bright staining.

*$^3H$-TdR incorporation assay*: HL-60 cells were cultured at a concentration of $5 \times 10^3$ cell/ml in 96-well plates (V-bottom) with or without IL-4. Cultures were assessed for $^3H$-thymidine ($^3H$-TdR) nuclear incorporation after 3 days of incubation. One µCi of $^3H$-TdR (of 20 Ci/mmol specific activity; NEN, Boston, MA) was added per well 4 h prior to harvest and then the cells were placed in scintillation fluid (toluen-omnifluor, NEN, 1.38 g/l) and counted in a beta counter (LKB, Finland).

*Statistical analysis*: In all experiments that were performed at least three times in triplicate, the Student's *t*-test was used for the evaluation of significance levels ($p$). All other statistical values show the mean ± standard deviation (SD).

### Results

*p34 as a candidate molecule for excessive cell growth*: The rapid proliferative potential of the human promyelocytic leukaemia cell line HL-60[5,6] has not so far been explained. Although many hypotheses have been formulated concerning soluble factors elaborated in its culture medium[6,15] no conclusive answer has yet been offered. It has been suggested, however, that a large panel of cells and cell lines, some of which are related to the HL-60 system in terms of lineage restriction, owe their uncontrolled growth to the product of the cdc2 gene, p34.[13] It was obvious then to inquire whether HL-60 also expressed such a product. By immunocytometric analysis it was shown that indeed the cells expressed constitutively almost 50% of surface $p34^{cdc2}$ (Table 1), a protein that is found on the cell's membrane as well as intracellularly.[13] In marked contrast, normal human monocytes, already differentiated and with no left capacity to proliferate without exogenous aid, showed no levels of $p34^{cdc2}$ expression whatsoever (Table 1). The conclusion from these two situations was that the anomalous growth of HL-60 could be caused by the p34 presence.

**Table 1.** Constitutive $p34^{cdc2}$ expression on human leukaemia cells may indicate a role for the cdc2 kinase in cellular growth

| Cell type | Percentage of positive cells (± SD) as assessed by immunofluorescence* |
|---|---|
| | $p34^{cdc2}$ |
| HL-60 | 49 ± 3** |
| KG-1a | 28 ± 2** |
| Normal monocytes | 2 ± 1 |

*As described in the Materials and Methods.
**Statistically significant compared with control value (normal monocytes).

*IL-4 as the link between $p21^{ras}$ and $p34^{cdc2}$*: Differentiation of HL-60 cells can be achieved after an appropriate chemical or physiological treatment.[6,8] For instance and for the sake of this work, IL-4 may drive these immature cells to express HLA-DR surface antigens, a marker of maturation.[8] Such induction has been shown by this laboratory to be G-protein ($p21^{ras}$)-dependent.[8] In addition, Pan *et al.*[9] have reported that in the *Xenopus* system oncogenic *ras* can block cell cycle progression by inhibiting $p34^{cdc2}$ activity. Therefore, since in our system induction of $p21^{ras}$ could be achieved by IL-4 treatment, the hypothesis formulated was that upregulation of this gene could be responsible for the differentiation IL-4 induced events via p34 modulation. The goal was to analyse the possible signals IL-4 could transmit to the HL-60 cells as to obtain a more mature progeny. As Table 2 shows, IL-4 indeed increases *ras* levels significantly ($p < 0.001$) and at the same time inhibits constitutive p34 expression ($p < 0.001$).

*Following the pathway*: In order to establish which event was first, co-culture of HL-60 cells with IL-4 and anti-$p21^{ras}$, a known inhibitor of *ras*,[8] was performed. It was shown that this combination could abolish the *ras* induction and restore the p34 expression, indicating that induction of *ras* precedes p34 modulation. According to the results of p34 on normal monocytes as described earlier, one could expect that downregulation of $p34^{cdc2}$ would bring cessation of cellular growth. Tritiated-thymidine incorporation assays demonstrated that this was exactly the case (Table 3). IL-4 could interfere at the abnormal proliferation rate of the cells by decreasing it significantly ($p < 0.05$). At the same time, HL-60 cells could be induced to express class II surface antigens as previously described.[8] To support the original conclusion that *ras* comes first as a signal transduced, it had to be demonstrated that inhibition of induced-*ras* (by the combination of IL-4 + anti-$p21^{ras}$ monoclonal antibody) could, except for restoring the suppressed p34 expression, inhibit the IL-4-induced HLA-DR upregulation. Indeed, blocking induction of *ras* reverted the cells to their initial class II negative phenotype.

**Table 2.** IL-4 induces $p21^{ras}$ and at the same time downregulates $p34^{cdc2}$

| Treatment | Percentage of positive cells* (± SD); day 3 | |
|---|---|---|
| | $p21^{ras}$ | $p34^{cdc2}$ |
| None | 5 ± 1 | 50 ± 3 |
| IL-4** | 24 ± 4*** | 25 ± 2*** |
| anti-$p21^{ras}$ | 1 ± 1 | 48 ± 5 |
| IL-4 + anti-$p21^{ras}$ | 11 ± 4**** | 53 ± 4**** |

*By immunofluorescence as given in the Materials and Methods.
**Doses and detailed experimental protocols are given in the Materials and Methods. Note that all assays were performed on day 3 (72 h) of culture, a timing that was best for class II induction.
***Statistically significant compared with control value (no treatment). *p* values are given in the text.
****Statistically significant compared with inducer value (IL-4). *p* values are given in the text.

**Table 3.** Cessation of growth is associated with HLA-DR upregulation

| Treatment | Percentage of positive cells* (± SD); day 3 | $^3$H-TdR* (cpm ± SD); day 3 |
|---|---|---|
| | HLA-DR | Proliferation |
| None | 6 ± 2 | 3020 ± 224 |
| IL-4 | 42 ± 6** | 987 ± 89** |
| anti-$p21^{ras}$ | 9 ± 1 | 2895 ± 138 |
| IL-4 + anti-$p21^{ras}$ | 11 ± 2*** | ND |

*Immunofluorescence and proliferation protocols are given in the Materials and Methods.
**Statistically significant ($p < 0.001$) compared with control value (no treatment).
***Blockade of DR induction by anti-$p21^{ras}$ antibody shows the sequence of events as described in the Results and Discussion sections.

### Discussion

IL-4 is an immunoregulatory lymphokine with multiple properties on immunological and haematological issues. Although it was first described as a co-factor for B-cell proliferation,[3] it was later shown to bind receptors on B- and T-lymphocytes, mast cells, macrophage,[16] and other types of cells.[4] Among its properties, one can briefly mention its role on (1) the regulation of B-cell growth, development and expression of membrane antigens; (2) its ability as a switch factor for IgE and IgG1 *in vitro* and *in vivo*; and (3) the regulation of T-cell growth and T-cell development in the thymus (for review see Paul[17]). In addition, one must not neglect its actions on a variety of haematopoietic cells, antitumour properties and proinflammatory involvement.[17–19] Recently, it has been shown that IL-4 may modulate gene expression for a number of colony-stimulating factors (CSFs) and interleukins like IL-1 and IL-6.[19]

Considering the above outlined properties, it has to be taken into account that the role of IL-4 is important for the initiation, maintenance and final yield of many regulatory mechanisms. Having the knowledge that IL-4 induces differentiation and class II antigen expression on HL-60 cells[8,18] and that the $p21^{ras}$ pathway is being activated by its presence in relation to class II upregulation, this study was undertaken in order to follow the possible intermediate pathways from the moment of IL-4 administration and until an autonomously grown cell, due to p34 expression, reaches a differentiated state.

The results presented in this study, not only support the hypothesis of Pan *et al.*[9] that *ras* may block cell cycle progression by p34 inhibition, but also point to the sequence of signals transduced by IL-4 on the HL-60 leukaemic population. It can be concluded that once IL-4 is added to the cells, an initial induction of *ras* is the major event responsible for the cascade that follows until the differentiation of this malignant clone. The IL-4 induced, *ras*-dependent inhibition of p34 appears to be responsible for the growth arrest, a very important step in the study of malignancy in general. Such inhibition brings an elevation of the maturation marker HLA-DR which shows that the cells have been programmed to differentiate and acquire a normal phenotype through this network of interactions that are reported for the first time for this particular factor and cells.

### References

1. von Boehmer H. The developmental biology of T lymphocytes. *Ann Rev Immunol* 1988; **6:** 309–326.
2. Adkins B, Mueller C, Okada CY, Reichert RA, Weissman IL, Sprangrude GJ. Early events in T-cell maturation. *Ann Rev Immunol* 1987; **5:** 325–365.
3. Paul WE, Ohara J. B cell stimulatory factor/interleukin-4. *Ann Rev Immunol* 1987; **5:** 429–459.
4. Park LS, Friend D, Sassenfeld HM, Urdal DL. Characterization of the human BSF-1 receptor. *J Exp Med* 1987; **166:** 467–477.
5. Collins SJ, Gallo RC, Gallagher RE. Continuous growth and differentiation of human myeloid leukemia cells in suspension culture. *Nature* 1977; **270:** 347–349.
6. Collins SJ. The HL-60 promyelocytic leukemia cell line: proliferation, differentiation and cellular oncogene expression. *Blood* 1987; **70:** 1233–1244.
7. Vassiliadis S, Kyrpides N, Papamatheakis J. The role of IL-4 in human myeloid leukemia: stimulation of RNA synthesis and transduction of differentiation signals through an IL-4 receptor leads to functional and HLA positive HL-60 cells. *Leuk Lymphoma* 1992; **7:** 235–242.
8. Vassiliadis S, Papamatheakis J. The p21$^{ras}$ protein as an intermediate signaling molecule in the IL-4 induced HLA-DR expression on normal and leukemic human myeloid cells. *Cell Immunol* 1992; **142:** 426–433.
9. Pan BT, Chen CT, Lin SM. Oncogenic ras blocks cell cycle progression and inhibits p34cdc2 kinase in activated *Xenopus* egg extracts. *J Biol Chem* 1994; **269:** 5968–5975.
10. Atherton-Fessler S, Parker LL, Geahlen RL, Piwnica-Worms H. Mechanisms of p34cdc2 regulation. *Mol Cell Biol* 1993; **13:** 1675–1681.
11. Meyerson M, Enders GH, Wu CL, Su LK, Gorka C, Nelson C, Harlow E, Tsai LH. A family of human cdc2-related protein kinases. *EMBO J* 1992; **11:** 2909–2914.
12. Grandin N, Reed SI. Differential function and expression of *Saccharomyces cerevisiae* B-type cyclins in mitosis and meiosis. *Mol Cell Biol* 1993; **13:** 2113–2119.
13. Streck RJ, Hurley EL, Pauly JL. Expression and capping of a proliferation-associated surface membrane p34kD antigen on different human hematopoietic cell lines. *J Leuk Biol* 1991; **50:** 182–191.
14. Athanassakis I, Galanopoulos VK, Grigoriou M, Papamatheakis J. Induction of class II MHC antigen expression on the murine placenta by 5-AzaC correlates with fetal abortion. *Cell Immunol* 1988; **128:** 114–121.
15. Fibach E, Peled T, Rachmilewitz EA. Self-renewal and commitment to differentiation of human leukemia HL-60 cells. *J Cell Physiol* 1982; **113:** 152–163.
16. Golb AE, Alm GV. Interleukin-4 down-regulates Sendai virus-induced production of interferon-α and -β in human peripheral blood monocytes *in vitro*. *Scand J Immunol* 1992; **35:** 167–175.
17. Paul WE. IL-4: a prototypic immunoregulatory lymphokine. *Blood* 1991; **77:** 1859–1870.
18. Vassiliadis S, Kyrpides N, Stravopodis D, Grigoriou M, Athanassakis I, Papamatheakis J. Investigation of intracellular signals generated by γ-interferon and IL-4 leading to the induction of class II antigen expression. *Med Inflamm* 1993; **2:** 343–348.
19. Donnelly RP, Crofford LJ, Freeman SL, Buras J, Remmers E, Wilder RL, Fenton MJ. Tissue-specific regulation of IL-6 production by IL-4. *J Immunol* 1993; **151:** 5603–5612.

**Received 30 June 1994; accepted 11 July 1994**

## References

[PDF_00338] Adkins B., Mueller C., Okada C. Y., Reichert R. A., Weissman I. L., Spangrude G. J. (1987). Early events in T-cell maturation.. Annu Rev Immunol.

[PDF_00358] Atherton-Fessler S., Parker L. L., Geahlen R. L., Piwnica-Worms H. (1993). Mechanisms of p34cdc2 regulation.. Mol Cell Biol.

[PDF_00344] Collins S. J., Gallo R. C., Gallagher R. E. (1977). Continuous growth and differentiation of human myeloid leukaemic cells in suspension culture.. Nature.

[PDF_00346] Collins S. J. (1987). The HL-60 promyelocytic leukemia cell line: proliferation, differentiation, and cellular oncogene expression.. Blood.

[PDF_00381] Donnelly R. P., Crofford L. J., Freeman S. L., Buras J., Remmers E., Wilder R. L., Fenton M. J. (1993). Tissue-specific regulation of IL-6 production by IL-4. Differential effects of IL-4 on nuclear factor-kappa B activity in monocytes and fibroblasts.. J Immunol.

[PDF_00370] Fibach E., Peled T., Rachmilewitz E. A. (1982). Self-renewal and commitment to differentiation of human leukemic promyelocytic cells (HL-60).. J Cell Physiol.

[PDF_00360] Meyerson M., Enders G. H., Wu C. L., Su L. K., Gorka C., Nelson C., Harlow E., Tsai L. H. (1992). A family of human cdc2-related protein kinases.. EMBO J.

[PDF_00355] Pan B. T., Chen C. T., Lin S. M. (1994). Oncogenic Ras blocks cell cycle progression and inhibits p34cdc2 kinase in activated Xenopus egg extracts.. J Biol Chem.

[PDF_00375] Paul W. E. (1991). Interleukin-4: a prototypic immunoregulatory lymphokine.. Blood.

[PDF_00364] Streck R. J., Hurley E. L., Pauly J. L. (1991). Expression and capping of a proliferation-associated surface membrane p34 kDa antigen on different human hematopoietic cell lines.. J Leukoc Biol.

[PDF_00348] Vassiliadis S., Kyrpides N., Papamatheakis J. (1992). The role of IL-4 in human myeloid leukemia: stimulation of RNA synthesis and transduction of differentiation signals through an IL-4 receptor leads to functional and HLA positive HL-60 cells.. Leuk Lymphoma.

[PDF_00377] Vassiliadis S., Kyrpides N., Stravopodis D., Grigoriou M., Athanassakis I., Papamatheakis J. (1993). Investigation of intracellular signals generated by gamma-interferon and IL-4 leading to the induction of class II antigen expression.. Mediators Inflamm.

[PDF_00352] Vassiliadis S., Papamatheakis J. (1992). The p21ras protein as an intermediate signaling molecule in the IL-4-induced HLA-DR expression on normal and leukemic human myeloid cells.. Cell Immunol.

[PDF_00336] von Boehmer H. (1988). The developmental biology of T lymphocytes.. Annu Rev Immunol.

